# A new auroral phenomenon, the anti-black aurora

**DOI:** 10.1038/s41598-021-81363-9

**Published:** 2021-01-19

**Authors:** A. E. Nel, M. J. Kosch, D. Whiter, B. Gustavsson, T. Aslaksen

**Affiliations:** 1 Centre for Space Research, North-West University, Potchefstroom, 2520 South Africa; 2 The South African National Space Agency, Hermanus, 7200 South Africa; 3 Department of Physics and Astronomy, University of the Western Cape, Bellville, South Africa; 4 Physics Department, Lancaster University, Lancashire, LA1 4YB UK; 5 Southampton University, Southampton, SO17 1BJ UK; 6 The University of Tromsø, 9010 Tromsø, Norway

**Keywords:** Space physics, Aurora

## Abstract

Black auroras are small-scale features embedded in the diffuse background aurora, typically occurring post-substorm after magnetic midnight and with an eastward drift imposed. Black auroras show a significant reduction in optical brightness compared to the surrounding diffuse aurora, and can appear as slow-moving arcs or rapidly-moving patches and arc segments. We report, for the first time, an even more elusive small-scale optical structure that has always been observed occurring paired with $\sim$ 10% of black aurora patches. A patch or arc segment of enhanced luminosity, distinctly brighter than the diffuse background, which we name the anti-black aurora, may appear adjacent to the black aurora. The anti-black aurora is of similar shape and size, and always moves in parallel to the drifting black aurora, although it may suddenly switch sides for no apparent reason. The paired phenomenon always drifts with the same average speed in an easterly direction. From the first dual-wavelength (427.8 nm and 844.6 nm) optical observations of the phenomenon recorded on 12 March 2016 outside Tromsø Norway, we show that the anti-black and black auroras have a higher and lower mean energy, respectively, of the precipitating electrons compared to the diffuse background.

## Introduction

Small-scale black aurora structures typically appear in the homogeneous diffuse aurora, defined as gaps within diffuse auroras (and sometimes between pulsating auroras)^1^. Although they appear black in the sky, optical emissions are present but have a lower luminosity relative to the surrounding diffuse aurora^2^. Black auroras are usually seen within the auroral oval during the late geomagnetic substorm recovery phase^3^, typically post-magnetic midnight. Several distinct forms have been observed, such as black patches, black arc segments, thin black arcs, and black vortex streets^1^ with a typical patch width of   0.5–4 km and length of  2.5–5 (up to 20) km at an assumed altitude of  105 km^3^. The average drift speed is typically  0.6–1.5 km s$^{-1}$ eastward (similar to pulsating auroras)^1^ and the observed drifts appear unrelated to the ionospheric plasma flow^4^. Black patches have generally no apparent shear-motion but black arcs may develop curls or vortices.

Two theories have been put forward to try and explain the existence of black auroras. One is the coupled ionospheric–magnetospheric generation mechanism that proposes that a downward field-aligned current is carried by cold electrons flowing out of the topside ionosphere within the black aurora and thus occurs in the ionosphere^5^. The other one is a magnetospheric generation mechanism which hinders scattering of high energy electrons into the loss cone within the black aurora^6^. Marklund et al.^5^ used Freja satellite electric field data but with no optical observations. Peticolas et al.^6^ used quasi-simultaneous ground-based optical and FAST satellite electron flux observations. Simultaneous measurements by the EISCAT incoherent scatter radar^7,8^ and a monochrome auroral TV camera were used to analyse several periods in which black aurora occurred^4^. The drift speed was compared to the estimated characteristic energy of each event and they concluded that the drift speed of these events were proportional to the energy of the precipitating electrons consistent with a gradient-B curvature drift, which points towards a magnetospheric mechanism for the black aurora^4^.

**Figure 1:**
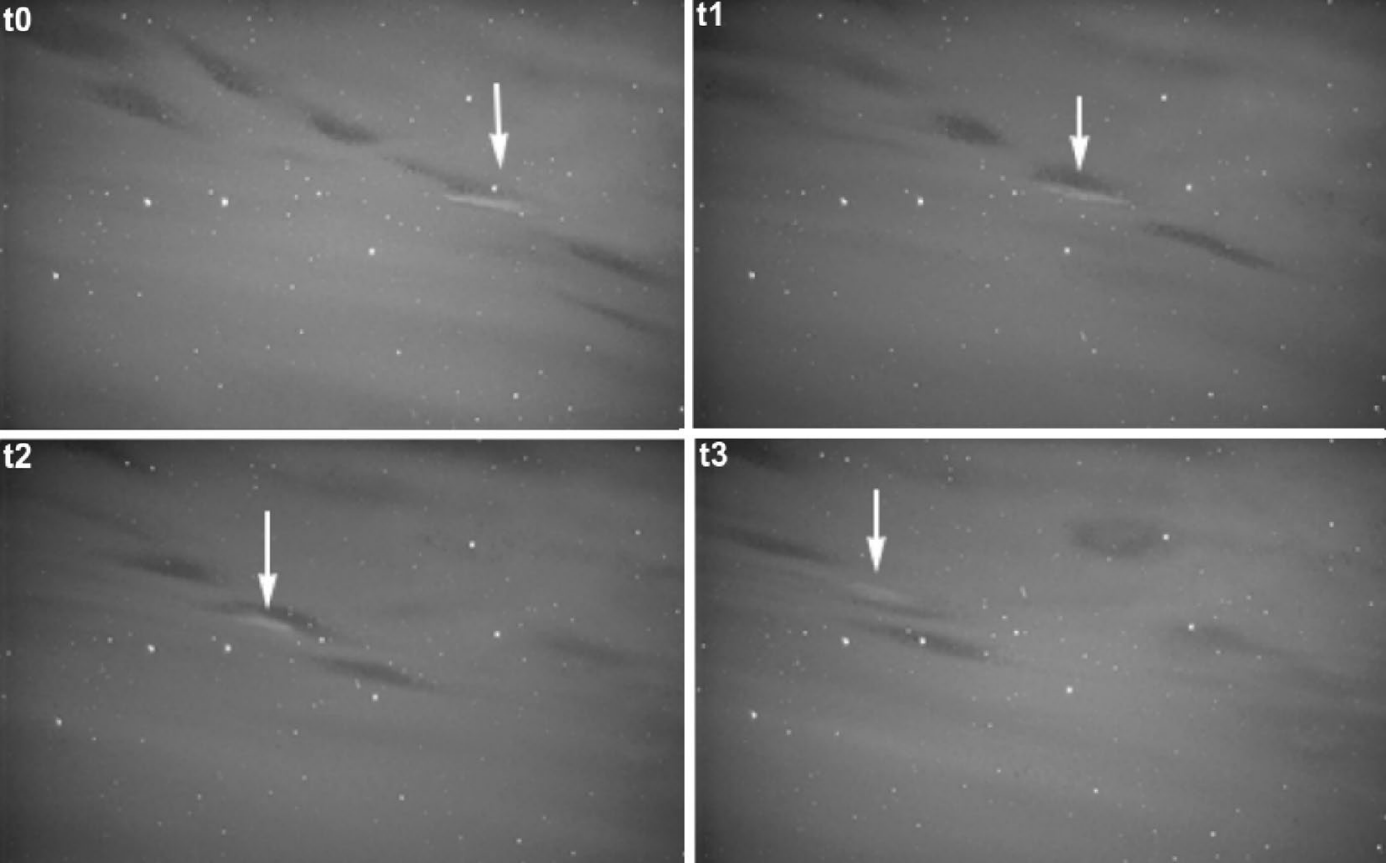
Time lapse white light images of the first anti-black aurora observed on 16 March 2007 from Tromsø, Norway, at about 21:30 UT. The greyscale or brightness range of the image is from black (dark) to white (bright). The frames have a $\sim \,20^{\circ }$ FOV based on the star field, and are in time steps of 1 s. North is up and East is left. (Dr. Torsten Aslaksen, Private communication). The white arrows highlight the black and anti-black aurora pair.

A chance observation on 16 March 2007 at around 21:30 UT, taken by a white-light TV camera from Tromsø, Norway, revealed the first definitive recording of a patch of enhanced luminosity drifting adjacent and parallel to a black aurora patch, as shown in Fig. 1. Note how the white patch is brighter than the diffuse background, through which the background starfield is clearly visible. Also, note that most black auroras in the camera’s field of view (FOV) do not have a discernible paired white patch. In addition, the white patch may “jump” to the opposite side of the black aurora, as seen in the final frame of the sequence. We name the white patch paired with the drifting black aurora as the anti-black aurora, mainly to distinguish it from the ordinary bright aurora and because we have only ever observed this phenomenon in association with moving black auroras. To the authors’ knowledge, no paired white patch drifting alongside a black aurora has been reported in the literature before.

Below we report on the first spectral optical observations of the anti-black auroras, recorded in 2016 at EISCAT near Tromsø, Norway, from which we infer the mean energy of the precipitating electrons simultaneously in the diffuse background, black and anti-black auroras for the first time.

## Instrumentation and observations

During the 2016 campaign, an iXon-888 EMCCD camera and an ALTA-U47 back-illuminated slow-scan bare CCD camera were mounted next to each other and ran in parallel during observations. EMCCD’s have eliminated the problem of read-out noise which makes low-light imaging with high frame rates possible^9^. With $2 \times 2$ pixel binning, the EMCCD produced images of 256 $\times$ 256 pixel resolution in a 30$^\circ$ FOV. It had a maximum temporal resolution of 10 Hz. The bare CCD produced the same resolution images in a FOV of 50$^\circ$, with a maximum temporal resolution of 1 Hz.

The EMCCD was fitted with an Andover 430HC10 optical filter (passband centre 430 nm, bandwidth 10 nm, passband transmission  90%) to observe the $N^+_2$ emission at 427.8 nm, which has an excitation threshold of $\sim$ 18.6 eV^10^. The bare CCD was fitted with an Andover optical filter (passband centre 845.6 nm, bandwidth 3 nm passband, transmission  85%) to observe the $\left( 2p^33p\right) ^3P \rightarrow \left( 2p^33s\right) ^3S$ transition of O at 844.6 nm, which has an excitation threshold of 11 eV^10^. The optical filters were chosen to be within the operating range of both cameras. Both chosen emissions are prompt allowed transitions, which is important because of the rapid motion and small size of the black aurora.

The EISCAT UHF incoherent scatter radar is located near Tromsø, Norway, with a fully steerable parabolic dish of 0.6$^\circ$ beam width^7,8^. The UHF radar can make high temporal (0.44 s) and high spatial (900 m range) observations of the plasma density profile parallel to the local magnetic field^4^ from below 100 to above 400 km range. This is important for estimating the energy spectrum of the precipitating electrons (discussed below). In addition, the radar routinely observes the electron and ion temperatures as well as ion velocity.

The two co-located cameras made simultaneous observations with the EISCAT UHF radar on 12 March 2016. In all cases, the images were produced with a frame cadence of 3s, a compromise between temporal resolution and signal-to-noise ratio. The EISCAT UHF radar ran the arc1 code, with a range resolution of 900 m and an integration time of 0.44 s, and was fixed pointing in the magnetic zenith direction ($\hbox {az} = 180^\circ$, $\hbox {el} = 77^\circ$). Observations ranged from 95.7 up to 422.4 km corresponding to electron energies less than $\sim$ 30 keV^11^. On 12 March 2016 during recording from $\sim$ 18:00 to 24:00 UTC, the $\hbox {K}_p$ index was 3$^+$ and 1$^-$, with an average Dst index of − 18 nT, and solar wind speeds of $\sim$ 500 km s$^{-1}$. Four groups of black and anti-black aurora pairs were clearly identified post-magnetic midnight (see Table 1) that drifted eastward close to the magnetic zenith, but unfortunately not directly into the EISCAT radar beam.

**Table 1:** Table showing the times (column 1) and drift velocities (column 2) of the selected anti-black aurora (ABA) and black aurora (BA) paired events recorded on 12 March 2016.

Event time (UT)	Drift velocity	Optical ratio inside black aurora (BA) and anti-black aurora (ABA)	Optical ratio inside diffuse aurora	Mean E inside BA and ABA (keV)	Mean E inside diffuse aurora (keV)
22:08:01–22:08:10	0.87 km s$^{-1}$ E	0.204 (BA), 0.175 (ABA)	0.177	4.8 (BA), 7.9 (ABA)	7.6
22:56:19–22:56:28	0.95 km s$^{-1}$ E	0.221 (BA), 0.165 (ABA)	0.187	3.3 (BA), 10.6 (ABA)	6.2
23:05:55–23:06:04	2.26 km s$^{-1}$ E	0.208 (BA), 0.165 (ABA)	0.173	4.6 (BA), 11.3 (ABA)	8.4
23:18:28–23:18:37	–	0.189 (BA), 0.162 (ABA)	0.178	5.9 (BA), 14.2 (ABA)	7.4

Only three events had measurable drift speeds. Optical ratios (I(O)/I($\hbox {N}_2^+$)) inside and outside the BA and ABA events are shown in columns 3 and 4, respectively. Likewise, the mean energies inside and outside the BA and ABA are shown in columns 5 and 6, respectively.

**Figure 2:**
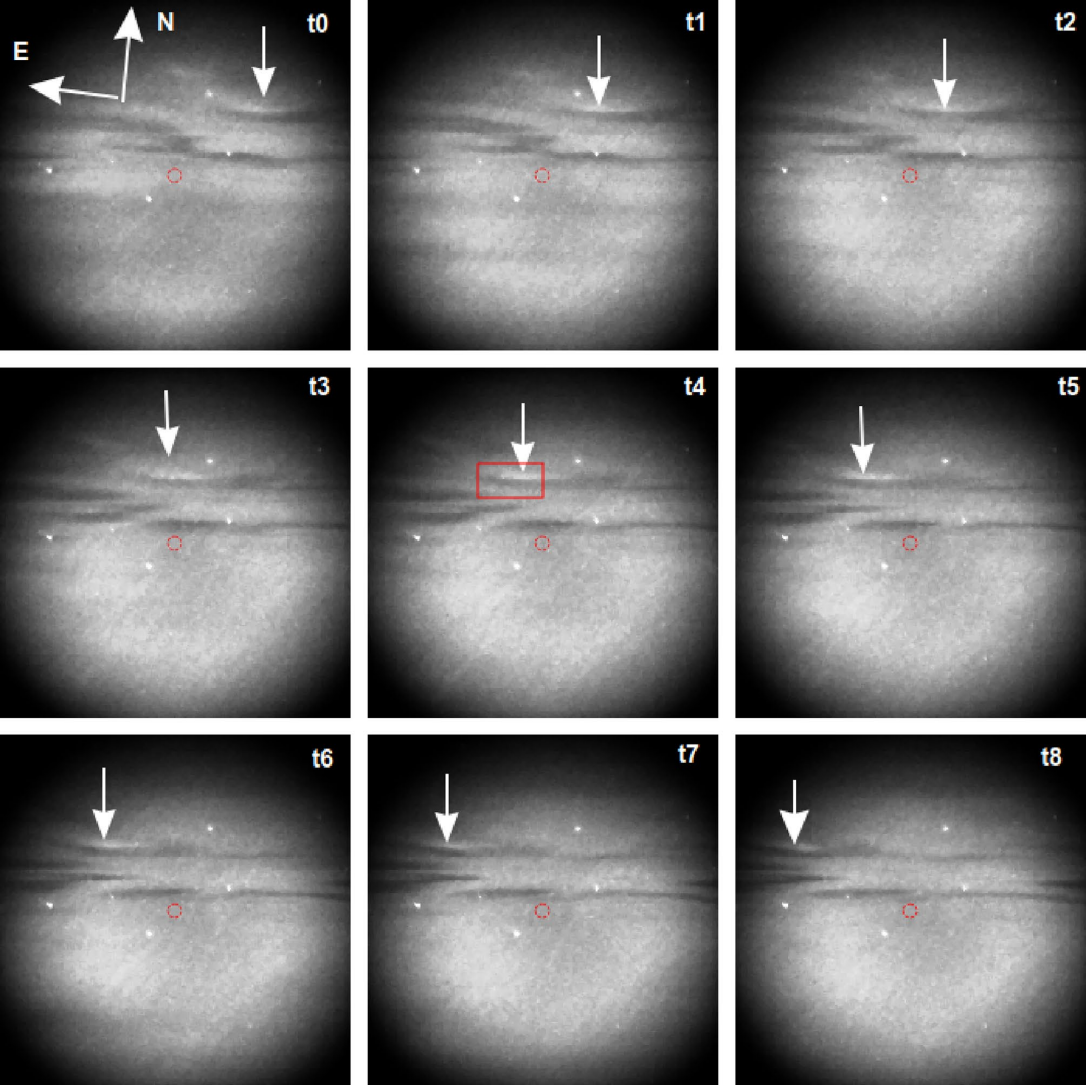
Time lapse of anti-black aurora event (arrow) on 12 March 2016 with the 427.8 nm filter from 22:56:10 to 22:56:34 UT with 3 s steps. The red circle indicates the EISCAT radar pointing direction. The red square denotes the area shown in Fig. 4. The greyscale/brightness range of the image is from black (dark) to white (bright).

**Figure 3:**
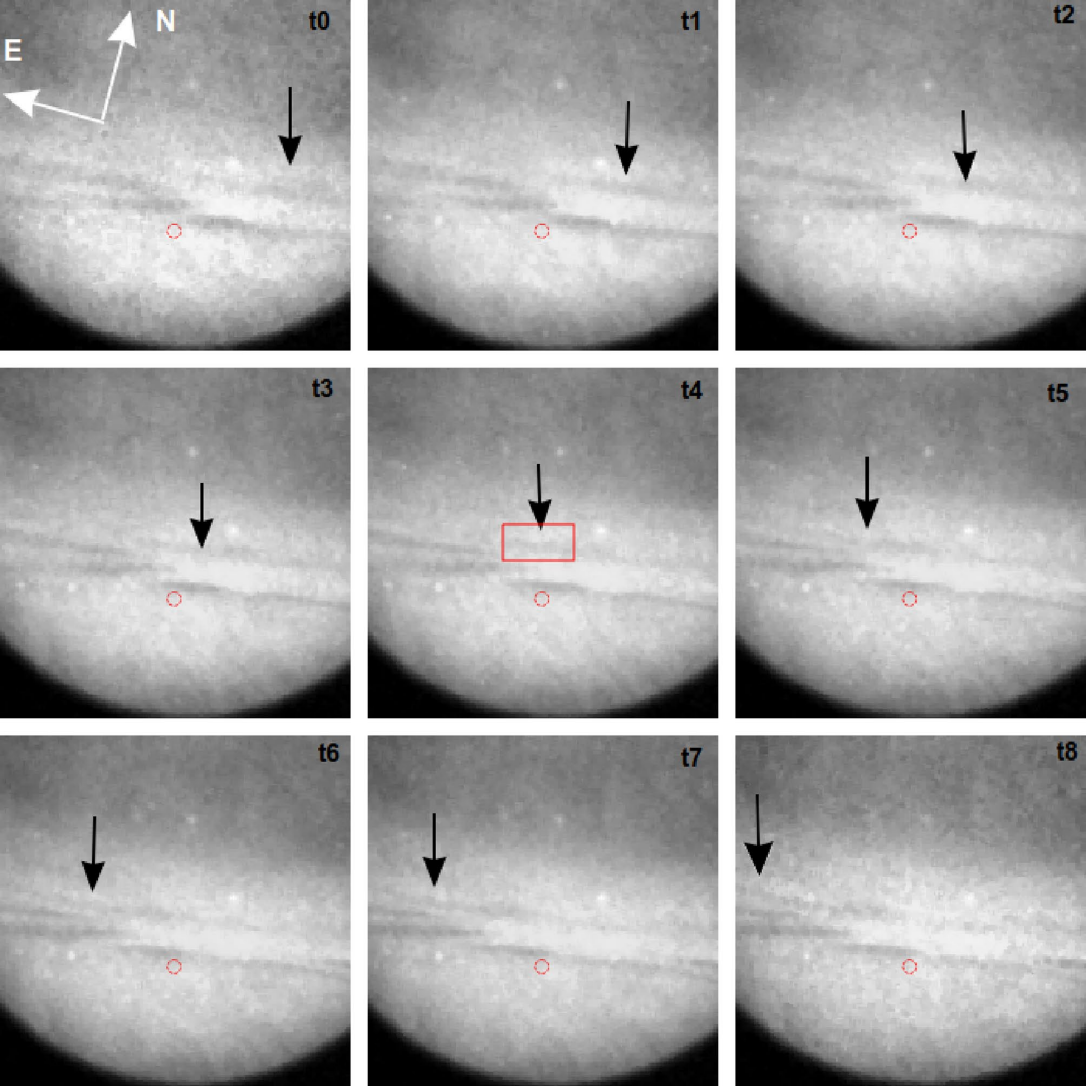
Same as Fig. 2 except for 844.6 nm filter and frames cropped.

Figures 2 and 3 show a time sequence from one event (22:56:10 to 22:56:34 UT) on 12 March 2016 using the 427.8 and 844.6 nm emission filters, respectively. Each figure shows 9 panels with 3 s time steps. The panels in Fig. 3 have been cropped to show the same FOV as in Fig. 2. The radar beam (red circle) mapped to 105 km in the magnetic zenith, and anti-black aurora (arrow) are demarcated. The white dots correspond to stars. It is important to note that the star field and background auroras may not appear identical in Figs. 2 and 3 due to the narrowband filters used. The dark corners in the images are due to vignetting of the wide-aperture optics, or the edge of the FOV. There is a clear systematic eastward drift of the black and paired anti-black auroras. In the 2016 data set reported here, we did not observe the anti-black aurora flipping to the other side of the black aurora. Although the 844.6 nm images show the same event, they have noticeably less contrast than the 427.8 nm images.

In addition to the morphology of the anti-black and black auroras, shown in Figs. 1, 2 and 3, a key parameter is the mean energy of the precipitating electrons creating the optical phenomenon. This can be determined uniquely by inverting the electron density altitude profile as observed by the EISCAT radar because new ionisation in the atmosphere depends on the flux of precipitating electrons from space whose final deposition altitude depends on their initial energy above the atmosphere^12^. The EISCAT arc1 code effectively covers all precipitating electron energies below $\sim$ 30 keV due to the altitude range covered when pointing into the (near vertical) magnetic zenith^11^. However, the radar only has a 0.6$^{\circ }$ FOV and the events analysed passed close by but never through the radar beam (see Figs. 2, 3). By assuming the energy spectrum of the precipitating electrons is a Maxwell–Boltzmann distribution^13^ we can use dual-wavelength optical image data, cross-calibrated against the radar data, to estimate the mean energy of the assumed spectrum anywhere in the FOV^2^. Due to the rapid motion of the anti-black and black aurora pairs, this can only be done using the prompt allowed auroral optical emissions. For the available camera technology, the most suitable emissions are 427.8 nm from $\hbox {N}_2^+$ at a relatively lower altitude (> 100 km) corresponding to a higher electron energy (< 10 keV), and 844.6 nm from O at a relatively higher altitude (> 180 km) corresponding to a lower electron energy (< 0.5 keV). The altitude difference of the optical emissions results in almost no ambiguity for our analysis as our observations were always close to the local magnetic zenith. The filtered optical data was calibrated into absolute energy flux units (10$^{-11}$ W m$^{-2}$) using the stars visible in the data frames^14,15^. The mean energy, taken from the optical data corresponding to the radar beam position in the sky (red circle in Figs. 2, 3), was cross-calibrated against the inverted radar data, following which the optical data were used to estimate the mean energy of the precipitating electrons elsewhere in the cameras’ common FOV.

## Results

The four anti-black aurora events analysed in this study are shown in Table 1. Listed are the time of each event, average drift velocity, and the respective optical ratios and mean energies of the black aurora, anti-black aurora, and the diffuse aurora background. The event observed at 23:18:28–23:18:37 UT was only partially visible, and tended to intermittently merge and split with other surrounding structures, thus the drift speed could not be accurately determined.

For an assumed altitude of 105 km^3^, the average horizontal width and length of the anti-black aurora was 2.1 (1.5) and 13.3 (4.4) km for the four events, respectively, where the values in brackets refer to the standard deviation. The paired anti-black auroras morphology is very similar to that of the black auroras. The mean drift speed of the anti-black aurora was 1.3 (0.8) km s$^{-1}$ eastward, which was practically identical to the black aurora. The ionospheric plasma convection velocity obtained from SuperDARN was near zero over the time period $\sim$ 22:00 to 23:30 UT, consistent with the low level of geomagnetic activity.

**Figure 4:**
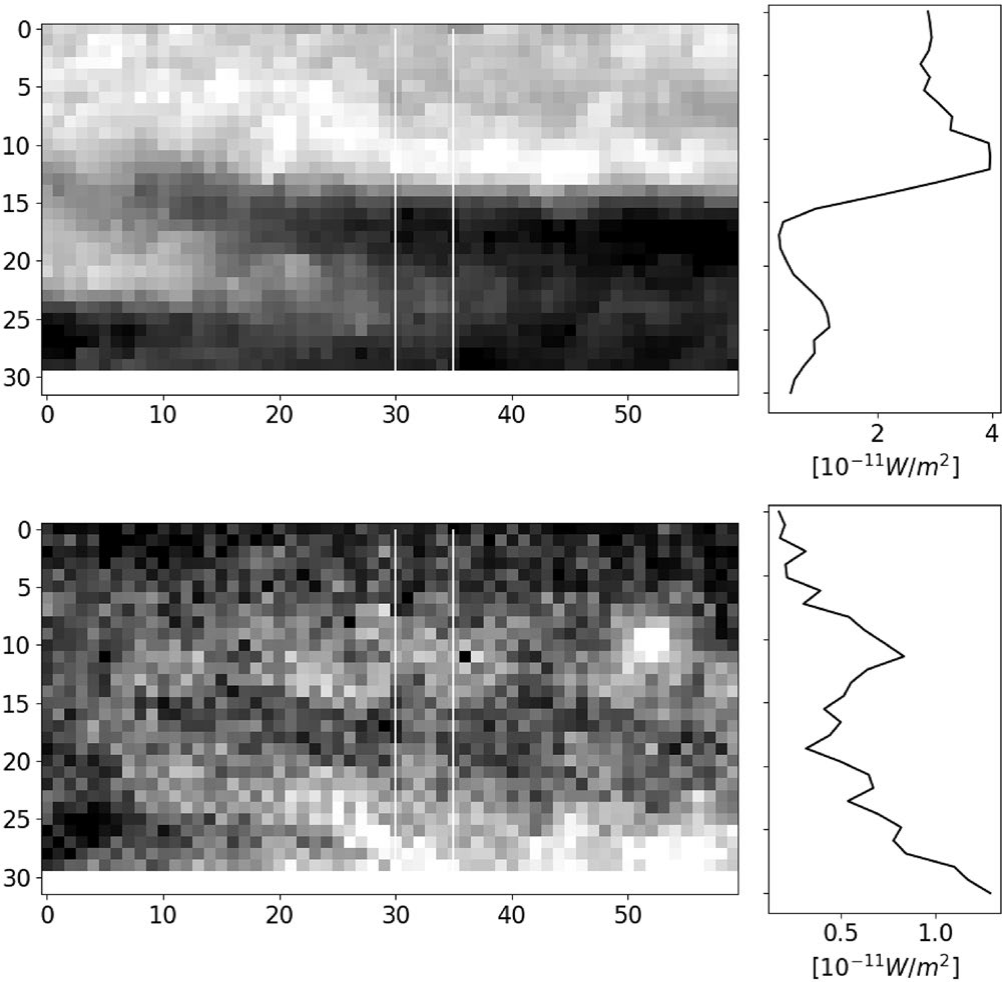
Zoomed image of anti-black/black aurora during 22:56:19–22:56:28 UT (panels t3–t5 of Figs. 2, 3, red squares in panel t4 show sampled location) for 427.8 (upper panel) and 844.6 nm (lower panel) with their respective calibrated brightness profiles (right-hand column) averaged between the solid white lines. The axes represent pixel coordinates.

Figure 4 shows the intensity profiles of one event (22:56:19–22:56:28 UT, panels t3–t5 of Figs. 2, 3). The intensity profile sampling lines (white) cross the black and anti-black aurora through their centres. This shows the anti-black and black auroras to be brighter and darker than the diffuse background, respectively. However, the intensity dip and peak are more obvious for 427.8 than for 844.6 nm. The optical intensity of the anti-black aurora was on average 10% and 6% brighter than the surrounding diffuse aurora in 427.8 and 844.6 nm, respectively, indicating an increase in the flux of the precipitating electrons for the four events. The greater increase for 427.8 nm (10%) than for 844.6 nm (6%) indicates an increase of the mean energy of precipitating electrons^2^. On the other hand, the optical intensity of the black auroras was on average 24% and 16% less than the surrounding diffuse aurora for 427.8 and 844.6 nm, respectively, indicating a decrease in the flux of the precipitating electrons for the four events. The greater decrease for 427.8 nm (24%) than for 844.6 nm (16%) indicates a decrease of the mean energy of the precipitating electrons^2^. This result for the black aurora is consistent with previous studies^2,16^ but that for the anti-black aurora is entirely new.

From the raw images, blocks of six pixels were selected to extract the mean energy of the precipitating electrons from the black, anti-black and diffuse auroras. By way of example, we present the averaged pixel values from the event at 22:56:19–22:56:28 UT on 12 March 2016 for both the 427.8 and 844.6 nm (see Figs. 2, 3). For 427.8 nm, the average raw pixel value for the anti-black aurora was 1410, the black aurora 1169, and the adjacent diffuse aurora 1355. The standard deviation is $\sim$ 33 data numbers. There were no clear background frames available during this evening, so a sample block was taken from a previous evening, 5 March 2016, using the same camera settings. Here the average background pixel value was 817 with a standard deviation of $\sim$ 37. These values show that we were clearly able to distinguish the three different types of auroras based on their intensities at 427.8 nm. For 844.6 nm, the average values were 1006 for the anti-black aurora, 955 for the black aurora, and 984 for the diffuse aurora. The standard deviation was $\sim$ 7 data numbers. The background sample from 5 March 2016 was 848 with a standard deviation of $\sim$ 15. Again, although with lower contrast and data values closer to the dark sky background, we were able to distinguish the different types of auroras based on their intensities at 844.6 nm. The mean value for the dark frames for the EMCCD (427.8 nm) was 94(9) and for the CCD (844.6 nm) 784(6) with standard deviation given in brackets.

**Figure 5:**
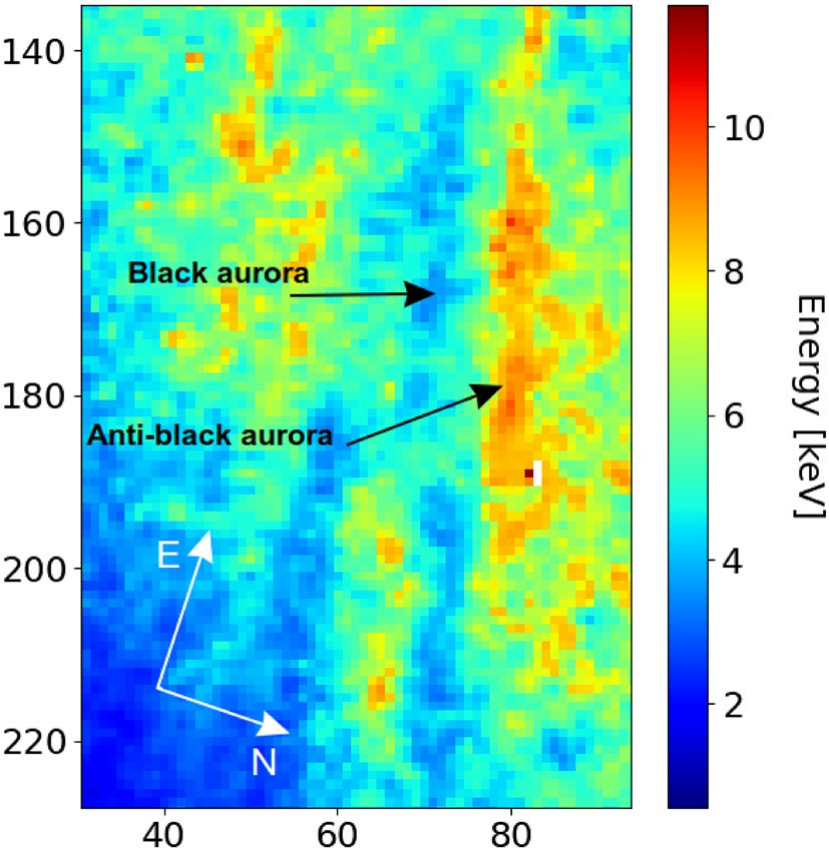
The mean energy of the precipitating electrons for 22:56:19–22:56:28 UT (panels t3–t5 of Figs. 2, 3), determined from the 427.8 and 844.6 nm optical data. The axes represent pixel coordinates.

Figure 5 shows the calibrated energy map of mean precipitating electron energy, extracted from the optical data, for the anti-black/black aurora pair (demarcated) on 12 March 2016 for 22:56:19–22:56:28 UT (corresponding to panels t3–t5 of Figs. 2, 3). It is clear that the anti-black aurora has a higher mean energy of the precipitating electrons compared to the black aurora. The mean energies are shown in Table 1 for all four anti-black/black aurora events observed on 12 March 2016. The average mean energy of the black, anti-black and diffuse background auroras was 4.8 (0.8), 11.0 (2.5), and 7.2 (1.1) keV, respectively. The figures in brackets are the standard deviation. The radar inversion uncertainty is $\sim$ 20%^12^ whereas the optical modelling uncertainty is $\sim$ 10%^17^. Previous studies have determined a mean of 4.4^16^ and 6.7 keV^4^ for black aurora energies, which is consistent with our findings. In our analysis, the mean energy of the precipitating electrons was 2.4 keV lower and 3.8 keV higher for the black aurora and anti-black auroras, respectively, than that of the surrounding diffuse aurora. These results are significant based on our uncertainty estimates. No other studies of the anti-black phenomenon exist currently for comparison.

## Discussion

We report the discovery of a wholly new type of aurora, namely, the anti-black aurora which is always paired with a drifting black aurora patch. The anti-black aurora presents itself as a bright patch or arc segment, of similar shape and size to the black aurora, that drifts alongside a black aurora at the same velocity in an easterly direction in $\sim$ 10% of all cases. The anti-black aurora has been observed to occasionally suddenly change to the opposite side of the black patch, apparently at random. The motion of the anti-black/black aurora pairs appears unrelated to the background ionospheric plasma convection velocity, consistent with previous observations of the black aurora^4^.

In addition, we present the first spectral optical information of this phenomenon, from which we determine that the mean energy of the precipitation electrons within the anti-black and black auroras is higher and lower, respectively, than that within the background diffuse aurora. This is consistent with previous knowledge regarding the black aurora but new for the anti-black aurora. Although the data set is small, it is interesting to note that the mean energy in the anti-black aurora is greater than the diffuse background by roughly the same magnitude as the black aurora is less than the diffuse background.

Previous studies have pointed towards a magnetospheric mechanism responsible for drifting black aurora events^4,6^. In the magnetosphere, electrons bouncing between the hemispheres drift eastward in longitude due to the magnetic field gradient and curvature in the equatorial plane^18^. Since auroras map along the magnetic field lines from the equatorial plane of the magnetosphere onto the ionosphere, their eastward drift velocity should also depend on the energy of the precipitating particles creating the auroras. This was found to be true for black auroras^4^. However, within our limited FOV, there was never any evidence that the anti-black aurora drifts faster than the black aurora despite its higher mean electron energy. We investigate this apparent contradiction further below.

From the ionospheric velocity, we can infer the energy of the electrons in the magnetosphere assuming pure gradient-B curvature drift. The magnetospheric drift speed $\hbox {v}_B$ is determined by mapping the observed black aurora zonal drift speed in the ionosphere out to the equatorial plane of the magnetosphere, using the Tsyganenko model^19^. By projecting back from the magnetospheric equatorial plane we can calculate the expected velocity of the anti-black aurora given its measured electron energy in the ionosphere and assuming a purely magnetospheric source mechanism. On this basis, the expected average velocity for the anti-black aurora for the 3 events in Table 1 would have been 2.2 km s$^{-1}$. Relative to the observed average velocity of 1.3 km s$^{-1}$ of the anti-black/black aurora pair, the average velocity difference of 0.9 km s$^{-1}$ would be easily observable within our FOV, amounting to $\sim$ 20 km horizontal distance or  40% of our FOV for a transit. This however is clearly not the case, as can be seen in Figs. 1, 2 and 3, with the velocity difference between the black and anti-black aurora being always too small to measure.

The lack of an observable drift velocity difference between the black and anti-black aurora patches might point towards a dual-mechanism, i.e. where the source particles undergo small-scale blocking of pitch angle diffusion for electrons at energies greater than 2 keV whilst moving eastward under gradient-curvature drift in the magnetosphere, as suggested by Peticolas et al.^6^ and supported by Gustavsson et al.^2^ for the black auroras, but that a simultaneous low-altitude paired deceleration and acceleration mechanism modifies the precipitating electron flux and energy to create the black and anti-black aurora pairs, respectively, as suggested by Marklund et al.^5^.

We measure the electron energy in the ionosphere after any possible low-altitude mechanism that might be present has acted and at low altitude the gradient-curvature drift mechanism is weak and so would be insensitive to a change in electron energy. Since this low-altitude mechanism can vary with time, and we do not know how, the energy we measure in the ionosphere does not relate well with the drift speed, at least not for anti-black auroras.

This combination of mechanisms could explain the systematic electron energy difference yet the same ionospheric drift velocity of the anti-black and black aurora pairs. This would also be consistent with the low-altitude intense electric fields observed by the Freja satellite, which may be associated with the black auroras^5^. However, we have no satellite observations to support this hypothesis at this time. This remains to be confirmed in a future study.

## Methods

Several processing techniques are applied to the raw optical data. Dark image subtraction is necessary to remove leakage current due to thermal electrons from pixels in CCD detectors^14^ and flat-fielding is used to reduce the effect of non-uniform lens transfer function on the image frames. The steps are summarised in the equation below,

1 $$\begin{aligned} F_{DN} = \frac{F_r - F_d}{F_f} \end{aligned}$$

where $F_{DN}$ is the corrected image with the intensity given in raw counts, also referred to as data number (*DN*), $F_r$ are the raw images, $F_d$ dark frames, and $F_f$ flat-field frames. To reduce the pixel noise of these frames, median spatial filtering is applied.

Star mapping was applied to verify the camera pointing direction for each observed event. This is necessary to correctly register the 427.8 and 844.6 nm images for calculating the precipitating electron mean energy. The Smithsonian Astrophysical Observatory Star SAO catalogue^20^ was used for this step. Bright stars are identified in the FOV, which were used to calibrate the image pixel data numbers into real flux units. The apparent photon flux of an object depends on its spectral output, as well as the spectral band of the filter. The effect of the filter passbands on the measured flux of the photons reaching the camera aperture needs to be taken into account^14^. This is done by convolving the passband, $T_{pass}\left( \lambda \right)$, of each filter with the incoming photon flux,

2 $$\begin{aligned} f_s = \iint f\left( \lambda \right) I_{AE} T_{pass}\left( \lambda \right) \,d\theta \,d\lambda \end{aligned}$$

where $f_s$ is the observed photon flux, $f\left( \lambda \right)$ is the spectral photon flux, and $I_{AE}$ is the atmospheric extinction correction factor. The atmospheric extinction correction can be omitted, because all the stars, as well as auroral events, are viewed near-vertical above the atmosphere and will suffer extinction equally. The spectral photon flux for each of these identified stars was obtained from the Pulkovo spectrophotometric catalogue^15^.

Only events close to the EISCAT radar pointing direction that present discernible boundaries between anti-black/black auroras and the background diffuse aurora have been analysed. For estimating the mean energy of the precipitating electrons (described below), a temporal average was taken over 3 frames (9 s) for each event in order to improve the signal-to-noise ratio. In addition, spatial averaging (typically 5 $\times$ 5 pixels) was used for the same reason to determine the photon flux within an auroral feature, i.e. anti-black/black auroras and diffuse background.

The energy of an auroral structure can be estimated in one of two ways. Ground-based optical techniques have been used extensively to study the effects of auroral energy deposition on the composition in the ionosphere^21^. By using photon emission rate modelling due to electron impact on the atmospheric constituents (O and $\hbox {N}_2$) for at least two different auroral wavelengths, the mean energy of the precipitating electrons for an assumed energy spectrum, typically the Maxwell-Boltzmann distribution^22^, can be determined. We have used the Southampton ion chemistry model, which is a time-dependent, one-dimensional model that estimates the energy of precipitating electrons using the intensity ratios of auroral emissions^23^. The model includes the variable $[O]/[N_2]$ atmospheric composition density ratio^16^ as a function of altitude and time, and more recently the $[O]/[N_2^+]$ ratio was also added to the model (Dan Whiter, private communication, 2018). The uncertainty is estimated to be $\sim$ 10%^17^.

The other method is to invert ionospheric electron density height profile data, measured by the EISCAT radar. This is based on the fact that the altitude of impact ionisation by precipitating electrons into the atmosphere depends on their initial energy above the atmosphere^12^. In this study we used the ELSPEC code^12^, which has the advantage that no assumption of the energy spectrum is required. ELSPEC also requires the atmospheric neutral and ion composition density as a function of altitude, which is derived from the MSIS^24^ and IRI models^25^. Uncertainty in the composition estimates may cause about 10% error in the total energy flux estimates^12^. The overall inversion uncertainty is $\sim$ 20%^12^. Given the small beam size (0.6$^{\circ }$), the chances of capturing an anti-black/black aurora with the radar are small, as was the case during the 2016 observational campaign. It is, however, useful to compare the mean energy estimated from within the radar beam with those obtained from the ion chemistry model for cross-calibration purposes, and then use the optical data for parts of the sky not covered by the radar beam.

## Data Availability

https://figshare.com/s/a518a315b3e63e3650d7.
